# Treatment of myelodysplastic syndromes in the era of precision medicine and immunomodulatory drugs: a focus on higher-risk disease

**DOI:** 10.1186/s13045-022-01346-9

**Published:** 2022-08-31

**Authors:** Razan Mohty, Rama Al Hamed, Ali Bazarbachi, Eolia Brissot, Arnon Nagler, Amer Zeidan, Mohamad Mohty

**Affiliations:** 1 Division of Hematology-Oncology and Blood and Marrow Transplantation Program, Mayo Clinic, Jacksonville, FL USA; 2 Department of Internal Medicine, Jacobi Medical Center/Albert Einstein College of Medicine, Bronx, NY USA; 3 Bone Marrow Transplantation Program, Department of Internal Medicine, American University of Beirut Medical Center, Beirut, Lebanon; 4 Department of Clinical Hematology and Cellular Therapy, Saint-Antoine Hospital, AP-HP, Sorbonne University, and INSERM, Saint-Antoine Research Centre, 75012 Paris, France; 5 Hematology and Bone Marrow Transplant Unit, Chaim Sheba Medical Center, Tel Hashomer, Israel; 6 Division of Hematology/Oncology, Department of Internal Medicine, Yale School of Medicine, Yale University, New Haven, CT USA

**Keywords:** Myeloid malignancy, High-risk MDS, Precision medicine, Targeted therapy, Immune dysregulation, Immunotherapy

## Abstract

Myelodysplastic syndromes (MDS) are a heterogeneous clonal disease of myeloid neoplasms characterized by ineffective hematopoiesis, variable degree of cytopenias, and an increased risk of progression to acute myeloid leukemia (AML). Molecular and genetic characterization of MDS has led to a better understanding of the disease pathophysiology and is leading to the development of novel therapies. Targeted and immune therapies have shown promising results in different hematologic malignancies. However, their potential use in MDS is yet to be fully defined. Here, we review the most recent advances in therapeutic approaches in MDS, focusing on higher-risk disease. Allogeneic hematopoietic cell transplantation is beyond the scope of this article.

## Introduction

Myelodysplastic syndromes (MDS) are a heterogeneous group of myeloid neoplasms characterized by ineffective hematopoiesis, variable degree of cytopenia, and an increased risk of progression to acute myeloid leukemia (AML). The annual age-adjusted incidence in the USA is approximately 4.0/100,000 persons, and the incidence substantially increases with age. Besides age, other established risk factors include male sex, obesity, smoking, and prior radiotherapy or chemotherapy exposure. Still, most cases remain idiopathic [1].

Despite the approval of five MDS-specific therapies in the USA since 2004 and the increasing use of allogeneic hematopoietic stem cell transplantation (allo-HSCT), the prognosis remains dismal for most patients with higher-risk (HR)-MDS. Data from the Surveillance, Epidemiology, and End Results (SEER) program, the authoritative source for cancer statistics in the USA, showed that patients with MDS in the USA have a relatively worse 5-year overall survival (OS) at 31.3% compared to some of the most common cancers (e.g., prostate (84.3%), breast (81.5%), non-Hodgkin lymphoma (61.6%), chronic myeloid leukemia (57.8%), and myeloma (40.7%)) [1]. It is therefore a cancer that could be aggressive in many patients and should be identified and managed as such.

One of the salient developments in MDS management, especially HR-MDS, is the introduction of hypomethylating agents (HMAs). In the landmark randomized AZA-001 trial [2], azacitidine (AZA) significantly improved the median OS to approximately 24 months compared to 15 months with traditional therapies. Based on these data, AZA has become a standard of care in managing patients with HR-MDS. However, replicating this success in the “real-life” setting has been challenging [3]. In reality, patients with MDS are older and have more comorbidities than in the clinical trial setting, including poor kidney function and poor organ reserve, resulting in worse outcomes than those observed when evaluating highly selected patients in clinical trials.

Furthermore, in another SEER registry analysis examining the survival of patients with refractory anemia with excess blasts (RAEB), the median OS improved only minimally from 11 (95% CI 10–13) months in the period from 2001 until 2003 to 14 (95% CI 13–15) months from 2007 until 2010. The latter period followed the approval of HMAs in the USA (AZA: 2004; decitabine (DEC): 2006). Although the International Prognostic Scoring System (IPSS) data are not available in this registry, RAEB, currently referred to as MDS with excess blasts (MDS-EB) in the WHO 2016 classification, was used as a proxy for HR-MDS [4]. Another way of looking at similar data is to calculate the percentage of patients with MDS surviving at two years by age at diagnosis and treatment year based on HMA availability. Again, OS has only marginally improved after HMAs availability compared to the pre-HMAs era [1].

More data to support the need for better treatment regimens beyond HMAs monotherapy are evident from several studies. A retrospective cohort study of 532 untreated RAEB patients aged ≥ 66 years at diagnosis identified from the SEER program compared AZA with DEC. There was no statistical difference in median OS being the 2 groups being 11 (95% CI 10–14) months for AZA versus (vs.) 12 (95% CI 11–16) months for DEC, *p* = 0.26 [5]. Yet, this study only included older patients. Another study of 632 HR-MDS patients of all ages (identified from the MDS Clinical Research Consortium), who received a median of 5 cycles of HMA (68% had received AZA), showed median OS from diagnosis of 17 (95% CI 15.8–18.4) months [6], substantially lower than that observed in the AZA-001 trial[2]. Outcome after AZA failure is even worse with a median OS of 5.6 months, as shown in a large analysis including over 400 HR-MDS patients [7].

While HMAs monotherapy improved some of patients' outcomes, CR occurs in only around 10–15% of them. Furthermore, it takes 4–6 months to reach a CR, many patients never achieve it, and most patients that do so lose it in 1–2 years [8]. The “real-life” median OS for patients with HR-MDS is probably closer to 15–17 months, much lower than the 24 months described in clinical trials [9]. New therapies are urgently needed to improve patient outcomes, and many studies are ongoing to address this unmet need. Still, the clinical trial development for HR-MDS, especially after HMA failure, has been quite challenging. Several reasons could explain this difficulty, the most crucial one being that HR-MDS is not one homogenous disease but a biologically and molecularly heterogeneous entity. While more than 85–90% of patients have ≥ 1 mutation, only 4 mutations are seen in > 10% of cases [10]. More than 45 mutations have been described, but none are specific to MDS, and the average number of mutations per patient is 2–4 [10]. Attempting to target all these mutations with the same therapy is very challenging. Another important aspect has been the poor understanding of resistance mechanisms, including primary *vs.* secondary failure, the mechanism of action of HMAs, and the inability to identify reliable predictors of response or lack of response. Moreover, most MDS patients are old, in their 70 s, and many have limited social support due to the death of a spouse and/or children living far away [11]. Also, they may live distant from tertiary centers, where trials are typically conducted, with transportation difficulties; all these factors have impeded trial participation and thus in part contributed to the lack of novel therapies [10, 12, 13].

Still, randomized clinical trials (RCT) have been performed, and there have been attempts to combine HMAs with other agents. The North American Intergroup Study SWOG S1117A showed similar overall response rate (ORR) with AZA plus lenalidomide or AZA plus vorinostat (a histone deacetylase inhibitor) compared to AZA alone and without any difference in OS (log-rank *p* = 0.68; log-rank *p* = 0.22, respectively) [14]. On the other hand, several phase II trials showing improved outcomes with combinations *vs.* AZA alone failed to show significant difference with combinations compared to AZA monotherapy in large RCTs. This has been true for the combination agents lenalidomide, vorinostat, volasertib, eltrombopag, romiplostim, pracinostat, and most recently with the monoclonal antibody (mAb) durvalumab [14–18]. Here, we review the latest advances in the management of high-risk MDS, focusing on high-risk disease.

## Precision medicine in MDS

The management of MDS follows a risk-adapted strategy [19]. Treatment schema has not changed dramatically since 2004, apart from the addition of luspatercept, a recombinant fusion protein that binds transforming growth factor β superfamily ligands to reduce SMAD2 and SMAD3 signaling pathways, and the approval of an oral version of decitabine for HR-MDS, at least in the USA. Luspatercept was approved for lower-risk (LR)-MDS patients with anemia and ring sideroblasts, refractory to erythropoiesis-stimulating agents, who are transfusion dependent (TD).

Despite the very few changes to the management schema over the last few years, the dawn of precision medicine or tailored medicine is emerging in MDS as it has with AML, both in the frontline and relapsed setting [20]. For example, the presence of *IDH* mutations (which will be discussed later) allows opportunities for targeted approaches. Germline mutations and mutations associated with bone marrow failures are also reported in some patients with MDS [21]. The problem is that compared with AML, *IDH* and *FLT3* mutations are relatively rare, but still, many novel agents are being tested for HMA-resistant/refractory MDS [12, 13], including (Table 1):

1. Molecularly targeted agents:
  *IDH1/2* inhibitors (ivosidenib, enasidenib, FT-2102)
  first-in-class mutant *p53* reactivator APR-246 (eprenetapopt)
  Splicing modulator H3B-8800
  *FLT3* inhibitors (e.g., gilteritinib)
2. Genetically agnostic small molecule inhibitors:
  pevonedistat
  venetoclax (VEN)
3. Immunotherapies:
  anti-PD-1/PD-L1 antibodies
  anti-CTLA4
  anti-TIM-3
  anti-CD47 antibodies
4. Chemotherapy/epigenetic agents:
  CPX-351
  Novel HMA (ASTX727, CC-486, guadecitabine)
  *HDAC* inhibitors

**Table 1:** List of trials assessing the use of targeted therapy in high-risk myelodysplastic syndrome

Drug	Mechanism of action and target	Combination	NCT	Patient population	Trial design	Outcomes	Status	References
Cedazuridine/decitabine	HMA with cytidine deaminase inhibitor	Monotherapy	NCT03306264	*N* = 133 MDS and CMML	Phase III (ASCERTAIN)	PKs equivalence between oral and IV AUC 98.9% ORR: 61.7% CR: 22% mOS: 31.7 m LFS: 29.1 m	Completed	[27, 29]
Cedazuridine/decitabine	HMA with cytidine deaminase inhibitor + BCL2 inhibitor	Venetoclax	NCT04655755	*N* = 9 HR-MDS and CMML	Phase I	ORR: 100% CR: 67% with venetoclax 200 mg and 17% with venetoclax 400 mg	Ongoing	[30]
Azacitidine	HMA + BCL2 inhibitor	Venetoclax	NCT02942290	*N* = 51 receiving RP2D Untreated HR-MDS	Phase Ib	ORR: 84% CR: 40% mDoR: 13 m Time to CR: 2.6 m mOS in pts with CR: 28.6 m	Ongoing	[39]
			NCT04401748	*N* = 500 Untreated HR-MDS	Phase III VERONA	No results yet	Ongoing	[40]
			NCT02966782	*N* = 37 R/R HR-MDS	Phase Ib	ORR: 39% CR: 7% mDoR: 8.6 m mOS: 14.8 m	Ongoing	[42]
APR-246	*TP53* inhibitor	Azacitidine	NCT03072043	*N* = 40 MDS with at least one *TP53* mutation	Phase II	ORR: 73% CR: 50%	Completed	[46]
		Azacitidine with or without APR-246	NCT03745716	*N* = 154 *TP53*-mutated MDS	Phase III	CR: 34.6% (APR-246) vs 22.4% (no APR-246)	Completed	[48]
Pevonedistat	Inhibitor of the NEDD8-activating enzyme	With or without azacitidine	NCT02610777	*N* = 120 HR-MDS, HR-CMML, and low-blasts AML	Phase II	ORR: 79% (comb) vs 57%, *p* = 0.065 CR: 52% (comb) vs 27%, *p* = 0.050	Completed	[53]
			NCT03268954	*N* = 454 HR-MDS, CMML, and low-blasts AML	Phase III (PANTHER)	CR: 24% (comb) vs 32% EFS: 17.7 (comb) vs 15.7 m, *p* = 0.557	Completed	[54]
Ivosidenib	*IDH1* inhibitor	Monotherapy	NCT02074839	Patients with hematologic malignancies *N* = 12 with R/R MDS	Phase I	ORR: 75% mDoR: 21.4 m	Ongoing	[55]
			NCT03503409	*N* = 26 (A): failed HMA (B): untreated (C): low-risk MDS	Phase II IDIOME	ORR = 69% (A: 54%, B: 91%) CR = 46% (A: 23%: B: 73%) mDoR = 7.4 m mOS = 14 m (A: 7.7 m; B: not reached)	Ongoing	[56]
Enasidenib	*IDH2* inhibitor	Monotherapy	NCT01915498	Patients with hematologic malignancies *N* = 17 with MDS	Phase I/II	Prior MDS treatment: ORR = 46% No prior MDS treatment: ORR = 75% (3/4) mDoR = 9 m mEFS: 11 m mOS: 16.9 m	Ongoing, close for recruitment	[57]
		With or without azacitidine	NCT03383575	*N* = 21, R/R MDS *N* = 25, untreated MDS	Phase II	ORR: 84% (comb) vs 43% CR: 24% both arms mOS 32.2 m (comb) vs 21.3 m	Ongoing	[58]
Emavusertib CA-4948	*IRAK4 inhibitor*	With azacitidine	NCT04278768	*N* = 43 R/R AML and HR-MDS	Phase I/IIa	Patients with MDS and SF3B1/U2AF1/FLT3 mutations: CR: 57% All patients without SF3B1/U2AF1/FLT3 mutations: CR: 1/29 (3.5%)	Ongoing	[72, 73]

HMA: hypomethylating agents; MDS: myelodysplastic syndrome; PKs: pharmacokinetics; AUC: area under the curve; ORR: overall response rate; CR: complete response; mOS: median overall survival; LFS: leukemia-free survival; HR: high risk; CMML: chronic myelomonocytic leukemia; mDoR: median duration of response; AML: acute myeloid leukemia; comb: combination; and EFS: event-free survival; m: months

CPX-351 has improved outcomes of patients with secondary AML, including those with history of MDS or AML with MDS-related changes [22]. In HR-MDS, CPX-351 also showed safety and efficacy in a phase II trial by the Groupe Francophone des Myélodysplasies. The study included 31 patients with intermediate- or high-risk MDS. The CR rate was 52%, 22/27 patients with initial blasts > 10% cleared blasts to < 5% after therapy, and 22/30 patients were able to receive allo-HCT [23].

The oral combination of decitabine and cedazuridine (C-DEC), a cytidine deaminase inhibitor, was approved in the USA in 2020 [24]. Decitabine is rapidly inactivated by the enzyme cytidine deaminase in the gastrointestinal tract and liver, reducing its oral bioavailability and preventing its use as an oral MDS therapy. Early phase clinical trials have shown that the addition of cedazuridine, which inhibits this degradation, is active and safe in early phase clinical trials with evidence of pharmacokinetic (PK) equivalence to intravenous (IV) decitabine. Following dose-finding studies [25, 26], a randomized cross-over phase 3 study (ASCERTAIN) was undertaken in 133 patients with MDS and chronic myelomonocytic leukemia (CMML), confirming this PK equivalence between IV and oral forms. The study met its primary endpoint with high confidence: oral/IV 5-day decitabine AUC 98.9% (90% CI 92.7–105.6) [27]. This study's clinical efficacy and safety results were updated in 2021 whereby the complete response (CR) rate was 22% (95%CI 15.1,29.8), marrow CR (mCR) 32.3% (*n* = 43), hematologic improvement 7.5% (*n* = 10) and the ORR (CR + partial response [PR] + mCR + HI) was 61.7% (95% CI 52.8–69.9). Thirty-four (26%) patients proceeded to allo-HSCT. After a median follow-up of 32 months, the median OS was 31.7 months (95% CI 28.0, NE), and the leukemia-free survival (LFS) was 29.1 months (95% CI 22.1, NE). This oral combination of C-DEC is the only oral HMA with systemic exposure equivalent to its injectable drug [28, 29].

A phase I study, presented at ASH 2021 meeting, assessed VEN and C-DEC combination in HR-MDS and CMML. Only 9 patients were treated. While response was seen in all patients, CR was observed in 2/3 patients with VEN 200 and 1/6 patients with VEN 400) [30]. While this is a small study with limited follow-up, it has opened the door for double oral therapy for management of HR-MDS. Clearly, longer follow-up of more patients as well as randomized studies is needed to assess the safety and efficacy of this approach. While both of these agents are available commercially in the USA, we would currently caution against the off-label use of this combination outside of clinical trial context. Ongoing studies of C-DEC at lower doses/shorter schedules are evaluating the value of this agent among lower-risk MDS patients as well (NCT04655755).

Another oral HMA agent that has been approved in the USA, but not for management of MDS, is the oral formulation of AZA CC-486, which is currently approved as a maintenance therapy for AML patients who have not been able to complete curative intent therapy after induction chemotherapy. In this setting among older patients, CC-486 has improved OS (24.7 months) compared with placebo (14.8 months), which led to the approval of this drug. CC-486 has also been tested in MDS in a randomized phase 3 trial of 216 patients with red blood cell (RBC) TD anemia and thrombocytopenia LR-MDS. This drug significantly improved rates of RBC transfusion independence (TI) for ≥ 56 days in 31% of patients compared to 11% with placebo (*p* = 0.0002). Similarly, RBC TI for ≥ 84 days was 28% and 6%, respectively, *p* < 0.0001. However, these results did not translate into an improvement in OS (not the primary endpoint) due to an increased risk of early death, mainly related to infections [31]. Based on this data, CC-486 was not approved in the USA for MDS management. Still, investigations are continuing, and the availability of both oral HMA agents could open the door to more novel oral combinations in the future for both AML and MDS.

### VEN and AZA

VEN is an orally bioavailable small proapoptotic molecule targeting BCL2 that releases proapoptotic proteins to induce apoptosis [32]. One of the common mechanisms of resistance to AZA is increased expression of BCL2; hence, the synergy between VEN and AZA demonstrated in preclinical data [33]. A lot of excitement has been generated in older patients with AML based on the results of the VIALE-A trial showing an improvement of the median OS with the AZA-VEN combination compared to the control group (14.7 months vs. 9.6 months, hazard ratio [HR] for death 0.66; 95% CI: 0.52–0.85; *p* < 0.001) [34]. A real-world retrospective study confirmed the safety and efficacy of AZA-VEN combination in patients with R/R advanced myeloid malignancies [35]. Therefore, there has been a lot of interest in testing VEN in HR-MDS.

Jilg et al. assessed, for the first time, with in vitro data using CD34^+^ BM-derived mononuclear cells (BMMNCs) from a cohort of MDS or secondary (s)AML patients (*n* = 21) the impact of VEN alone or in combination with AZA on these cells. Low-dose AZA was as effective as high dose in reducing primary malignant MDS/sAML cells. In addition, malignant cells were targeted while sparing healthy hematopoiesis, even after HMA failure (*n* = 13) [33, 36, 37].

The open-label, dose-escalation, phase Ib single-arm study (NCT02942290) evaluated VEN and AZA for treatment-naïve HR-MDS. Results were presented at ASH 2020 meeting then updated at the ASH 2021 meeting. Seventy-eight patients aged ≥ 18 years, IPSS ≥ 1.5, BM blasts < 20% at baseline, and an Eastern Cooperative Oncology Group (ECOG) score ≤ 2 were enrolled. Patients with CMML, therapy-related MDS, and candidates for intensive chemotherapy or allo-HSCT were excluded. Unlike the 28-day dosing in AML, these patients were given VEN every 14 days. Doses were 100 mg (*n* = 8), 200 mg (*n* = 9), or 400 mg (*n* = 8), and the latter was eventually chosen as the RP2D (*n* = 51). For the 51 patients receiving RP2D, the median follow-up was 23 months. High efficacy was observed, with an ORR of 84%, 40% were CR, and the median duration of response (DoR) was 13 months (95%). Time to CR was relatively short at 2.6 months, compared to the 4–6 months commonly observed for HMAs [38]. Clinical responses (CR + mCR) were observed across all mutations, including TP53 (83%), ASXL1 (82%), and RUNX1 (71%). Grade 3 or 4 adverse events were observed in 96% of the patients with neutropenia (84%), including 45% febrile neutropenia and 42% thrombocytopenia. Median OS was 28.2 mOS (95% CI 17.7, NR). Median OS for 31 patients achieving CR was 28.6 months [39]. The phase III randomized VERONA trial that is ongoing comparing AZA/placebo vs. AZA/VEN will provide the definitive answer regarding using this combination (NCT04401748) [40].

The relapsed/refractory (R/R) MDS setting is associated with high risk and poor survival, and safety and efficacy of the combination of VEN and AZA is being evaluated in a similarly designed phase Ib open-label, multicenter study (NCT02966782) of 44 patients with R/R MDS. As clinical benefits from VEN monotherapy were limited, the investigators focused on the combination of VEN with AZA. Updated results for the 37 patients evaluable for response were presented at the ASH 2021 meeting. VEN was used for 14 days only, and AZA was used in the standard 7-days schedule. The median follow-up was 21.2 months (range, 04–37.5). The ORR (CR + mCR) was approximately 39%, and while most were mCRs, 7% had CR, and their median DoR and OS were 8.6 (6.0–13.3) and 14.8 months (95% CI: 11.3 – not estimable), respectively. mCR + HI were 6/14 (43%) patients. TI (RBC or platelets) was achieved by 10/32 (31%) of patients who were TD at baseline. These findings compare favorably with historical OS rates of around 6 months [41]. Safety data are in line with what was shown in the frontline setting [42]. Nevertheless, few conclusions can be drawn from this single-arm study of relatively small size, and data should be confirmed in a large, randomized trial.

### APR-246 in HR-MDS

*TP53* mutations are seen in around 20% of MDS and AML and are detected in 30–40% of patients with therapy-related disease. CR rates are very low approximately 15–20% in patients carrying these mutations, and efforts are directed toward targeting these mutations. APR-246 is a prodrug that is spontaneously converted to methylene quinuclidinone which induces apoptosis in *TP53*-mutated malignant cells [43]. Also, it increases oxidative stress promoting cell death. Preclinical studies have shown activity of APR-246 and synergetic effect with AZA in vitro and in vivo in *TP53*-mutated tumors [44]. Following these results, a phase I trial using eprenetapopt (APR-246) monotherapy showed activity through the activation of TP53-dependent pathways [45]. This was followed by a multicenter phase 2 trial using eprenetapopt in combination with AZA for patients mutated*TP53* MDS and oligoblastic AML. One hundred patients were included including 74 patients with MDS. Nausea and vomiting were the most common adverse events (58%) followed by febrile neutropenia (37%). Neurologic events including ataxia (26%) and dizziness (23%) were observed and were all reversible. The ORR was 69% including 43 CR. Measurable residual disease (MRD) negativity by next-generation sequencing was observed in 40 patients. After a median follow-up of 28 months, the mOS was 11.8 months. These results show that APR-246 and AZA combination is well tolerated and associated with high response rates [46, 47]. The phase 3 trial assessing this combination showed higher CR rate with the combination (34.6%) compared to azacitidine alone (22.4%) (NCT03745716) (48). Publication of these results is awaited.

### Pevonedistat in HR-MDS

Another drug that has generated excitement is pevonedistat, a first-in-class inhibitor of the NEDD8-activating enzyme; this inhibition blocks ubiquitination of select proteins upstream of the proteasome [49, 50], leading to disruption of the cell cycle progression and cell survival, with selective pre-apoptosis in some cancers [50, 51]. Pevonedistat has exhibited synergistic activity when combined with HMAs (AZA and decitabine) in cellular and mouse xenograft models of AML [52]. A randomized phase II study was initiated in 120 patients comprising HR-MDS (*n* = 67), HR-CMML (*n* = 17) and low-blast AML (*n* = 36). They were randomized 1:1 to either pevonedistat + AZA or AZA alone [53]. Patients had not previously received HMA therapy and were ineligible for allo-HSCT. The primary endpoint was originally event-free survival (EFS, defined as time to death or transformation to AML) but was changed to OS based on regulatory feedback. There was minor improvement in median EFS with 21 months versus 16.6 months for the combination of pevonedistat and AZA *vs.* AZA alone (HR = 0.665, 95% CI 0.423–1.047; *p* = 0.076) [53]. EFS and OS were not significantly different between the two subgroups in the subgroup analysis of only 67 HR-MDS patients (P-2001, NCT02610777); however, the analysis was not powered to look at this subgroup. Interestingly, the ORR and CR rate were higher in the combination therapy at 79% and 52% compared with 57% and 27% in the AZA monotherapy group, however, not statistically significant (*p* = 0.065 and *p* = 0.050), respectively [53]. Based on these results, a phase 3 randomized trial (PANTHER) was conducted [54]. It included 454 patients with HR-MDS/CMML or low-blast AML. The study unfortunately did not achieve its primary endpoint of improvement in EFS with 17.7 months in the combination vs. 15.7 months in AZA alone arms, *p* = 0.557. In HR-MDS patients, the CR rate in AZA alone was surprisingly even higher than the combination (32% vs. 24%, respectively) [54].

### Targeting *IDH1* mutation in patients with MDS

Ivosidenib (IVO) is a targeted inhibitor of the mutant *IDH1* (m*IDH1*) enzyme, another agent that has been studied in MDS. It was approved for use in AML. *IDH1* mutations, although not as common in MDS as compared with AML, are present in about 5–10% of cases. In a phase 1 dose-escalation and expansion study of IVO in m*IDH1* advanced hematologic malignancies, 12 patients with R/R MDS received IVO monotherapy. Median age was 72.5 years (range 52–78). Patients received IVO for a median of 11.4 months (range 3.3–42.5). Nine (75%) of the 12 patients had received prior HMA therapy. The ORR was 75.0% (95% CI 42.8–94.5) with a median DoR of 21.4 months (95% CI 2.3-NR). Nine patients (75.0%) were TI for ≥ 56 days during study treatment [55]. Based on these results, the FDA granted a Breakthrough Therapy Designation status for IVO monotherapy in this indication, and the study was amended to enroll additional m*IDH1* R/R MDS patients aiming to provide further understandings on safety, tolerability, clinical activity, and PKs/pharmacodynamics of treatment with IVO in approximately 23 patients (from the USA and France) with m*IDH1* R/R MDS. Another study assessing the use of IVO in *mIDH1* MDS is the IDIOME study, and results were presented at the 2021 ASH meeting. It included 32 patients, of which 26 were evaluable for the primary endpoint of ORR. The study included 5 and 13 patients with HR and very HR-MDS. The ORR was 69%, and 46% of the patients achieved CR. The median DoR was 7.4 months, and the median OS was 14 months. The most common side effect was differentiation syndrome [56].

### Targeting *IDH2* mutations in patients with MDS

Similar data were generated with the *IDH2* inhibitor, enasidenib (ENA). A subgroup analysis was carried out for patients with *IDH2*-mutated (*mIDH2*) MDS (which occurs in 5–10% of patients with MDS) in the phase I dose-escalation and expansion part of the multicenter, open-label, phase I/II AG221-C-001 trial of patients with advanced hematological malignancies. Seventeen patients (median age 67 years) with MDS were treated with ENA. Thirteen (76.5%) had received prior HMA therapy, and 6 (46%) achieved a response, some of which were durable. Of the 4 patients with no prior MDS treatment, 3 responded (1 PR, 2 mCR). The time to first response was 1.2 months, and the median DoR was 9 months. The median OS was 16.9 months, and median EFS was 11 months [57]. Enasidenib is generally well tolerated and is being considered for off-label use for patients with HR-MDS who fail HMA monotherapy or as combination therapy in the frontline setting. Preliminary results from the phase II study by the MD Anderson Cancer Center (MDACC) [58, 59], evaluating the efficacy and tolerability of ENA alone (*n* = 21) and in combination with AZA [treatment naïve] (*n* = 25) in *mIDH2* HR-MDS, showed promising efficacy. The ORR was 84% in the combination arm and 43% in the monotherapy arm. The CR rate is 24% in both arms. After a median follow-up of 12.6 months, median OS was 32.2 months in the AZA + ENA arm and 21.3 months in the ENA group [58]. The study is ongoing and continues to accrue (NCT03383575). ENA was also tested in IDH2 mutated MDS in the Ideal phase 2 study by the GFM group. Patients with HR-MDS were included in cohort A and B, and those with LR-MDS in cohort C. Cohort B allowed the addition of AZA in non-responders after 3 cycles [60]. Of 45 patients included, 26 were evaluable. ORR was achieved in 11 patients with 6 patients achieving CR. In cohort B, AZA was added to ENA in 3/9 patients. After a median follow-up of 8.6 months, the mOS was 17.3 months. Three patients experienced differentiation syndrome. Diarrhea and thrombocytopenia were the most common grade 3–4 side effects observed on 4 and 5 patients, respectively [60]. The study is still ongoing (NCT03744390). While not yet approved by the FDA, ENA can be used off-label for patients after HMA failure who cannot have an allo-HSCT or participate in a clinical trial.

### *FLT3* mutation in MDS

*FLT3* mutations in MDS occur at a lower frequency than in AML (0.6–6%) [61]. In a retrospective review by the MDACC, *FLT3* mutation analysis performed on 1232 MDS patients identified 12 (0.95%) such mutations [62]. A phase I/II study included patients with AML or HR-MDS ineligible to intensive chemotherapy to receive midostaurin, a broad-spectrum tyrosine kinase inhibitor, with AZA. After a median follow-up of 12 weeks, the ORR was 26% in all patients, and only 2% achieved CR [63]. Several studies are ongoing evaluating the use of gilteritinib and quizartinib in patients with AML and HR-MDS (NCT04027309, NCT04140487, NCT03661307, NCT04493138, NCT01892371).

### Splicing modulator H3B-8800

RNA spliceosome somatic mutations have been described in myeloid malignancies with SF3B1, SRSF2, U2AF1, and ZRSR2 being the most common [64]. These mutations lead to alternative mRNA splicing causing aberrant transcripts causing defective erythropoiesis and variable cytopenias leading to different spectrum of myeloid disease [64–66]. Therefore, targeting these mutations was testing in MDS. H3B-8800 is a splicing modulator showing therapeutic potential in spliceosome-mutant cancers in preclinical studies [67, 68]. In a phase I first-in-human trial H3B-8800 was studied in myeloid malignancies. Forty-two patients with MDS were included with 21 patients having high-risk disease [69] (NCT02841540). No remissions were observed. Yet, of 15 patients with MDS carrying SF3B1 mutation, 5 became transfusion independent. These findings show that H3B-8800 is safe and can lead to transfusion independence.

### IRAK4 inhibitor

RNA splicing factor mutations occur in around 50% of patients with MDS, with isoform expression of interleukin-1 receptor-associated kinase (*IRAK4*) being the most frequently identified alteration. Preclinical studies showed that *U2AF1* and *SF3B1* mutations lead to increased expression of the long isoform of the protein (*IRAK4-L*) in patients with AML and MDS [70, 71]. Additionally, high levels of *IRAK4-L* expression was associated with increased risk of progression of MDS and worse prognosis [70]. These findings led to the evaluation of a first-in-class oral inhibitor of *IRAK4*, emavusertib (CA-4948), in patients with R/R AML or HR-MDS in a phase I/IIb trial. A total of 49 patients were enrolled in the phase I dose-expansion study and received CA-4948 as monotherapy or in combination with AZA-VEN. Patients with *SF3B1*, *U2AF1*, or *FLT3* mutations demonstrated better response with 4/7 patients with MDS achieving marrow CR. In patients without *SF3B1/U2AF1/FLT3* mutations, only 1 patient (of 29 total patients) achieved CR. CA-4948 was safe without any dose-limiting toxicity [72, 73]. The trial is ongoing (NCT04278768), and further follow-up and larger studies are needed to assess the efficacy of emavusertib in HR-MDS. In April 2021, the FDA granted orphan drug designation to CA-4948 for treatment of AML and MDS.

## Immune dysregulation in myeloid malignancies

**Fig. 1:**
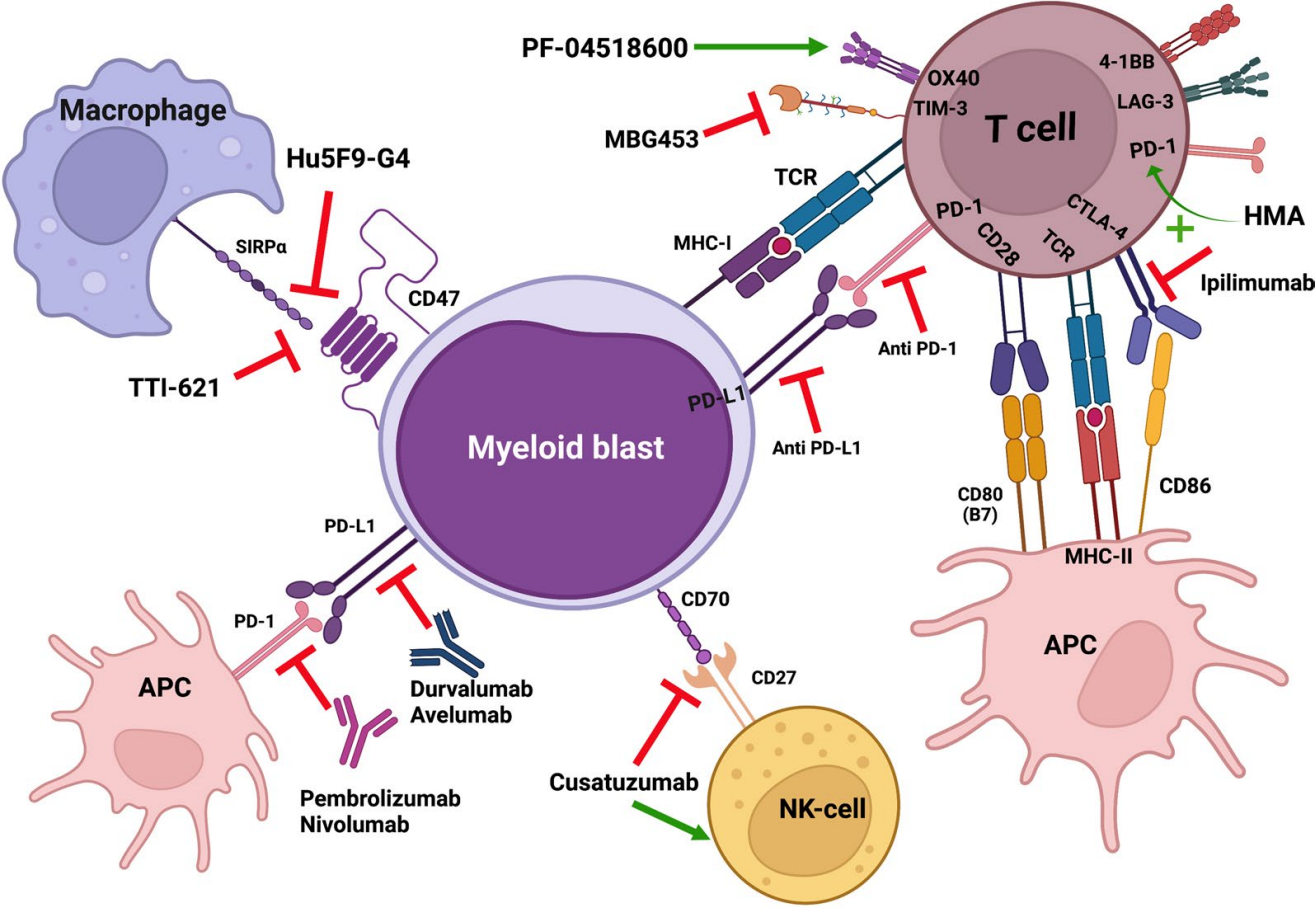
Boosting immune response through blocking inhibitory pathways and activating immune cells. HMA: hypomethylating agents; APC: antigen-presenting cell; MHC: Major histocompatibility complex. Created with BioRenders.com

Another primary focus in treating myeloid disease, in particular MDS, is immunotherapy (Table 2). Allogeneic hematopoietic cell transplantation is the most potent anti-MDS therapy, and currently the only modality with curative potential, but is beyond the scope of this review. Multiple immune aberrations and dysregulations exist in AML and HR-MDS leading to an immunosuppressed microenvironment with T cell exhaustion and senescence. Dysfunction of effector T cells, increased expression of coinhibitory molecules and tolerogenic dendritic cells, increased regulatory T cells (Tregs) [74], and dysfunctional and deficient natural killer (NK) cells lead to exhaustion and weakened immune response against malignant cells (Fig. 1). Immunosuppressive therapy, including cyclosporine and anti-thymocyte globulin combination, has shown efficacy is some type of low-risk MDS, especially in hypoplastic MDS or those associated with bone marrow failure syndrome [75]. Comprehensive immunologic studies on paired pre- and post-chemotherapy samples have revealed that the transcriptional and phenotypic T cell footprint response distinguishes responders from non-responders. In addition, in-depth analysis reveals alterations in multiple genes encoding cosignaling molecules that regulate immune responses [76].

**Table 2:** List of trials assessing the use of immunotherapy in high-risk myelodysplastic syndrome

Drug	Mechanism of action and target	Combination	NCT	Patient population	Trial design	Outcomes	Status	Reference
Ipilimumab	Anti-CTLA4 monoclonal antibody	Monotherapy	NCT01822509	*N* = 28 with hematologic malignancies *N* = 2 with MDS	Phase I	1 patient with MDS transformed to AML	Completed	[80]
		Monotherapy	NCT01757639	*N* = 29 HR-MDS Failed HMA therapy	Phase I	mOS: 9.8 m	Completed	[79]
Ipilimumab or Nivolumab	Anti-CTLA4 or PD-1 monoclonal antibody	With or without azacitidine	NCT02530463	MDS Treatment naïve: *N* = 41 HMA failure: *N* = 35	Phase II	ORR/CR: Nivo + AZA: 75%/50%; Ipi + Aza: 71%/38%; Nivo: 13%/0%; Ipi: 35%/15%	Ongoing	[81]
Pembrolizumab	Anti-PD-1 monoclonal antibody	Entinostat	NCT02936752	MDS, after HMA failure	Phase Ib	No results yet	Ongoing	[108]
Durvalumab	Anti-PD-L1 monoclonal antibody	Arm A: azacitidine Arm B azacitidine with durvalumab	NCT02775903	Untreated HR-MDS population *N* = 84	FUSION-AML-001	ORR: 67% arm A vs. 47.6% arm B; *p* = 0.18 mOS: 11.6 m (A) vs. 16.7 m (B); *p* = 0.74	Completed	[83]
Lenalidomide	Immunomodulatory drug	15 mg or 50 mg	NCT00867308	R/R HR-MDS and AML with MRC	Phase II	ORR: 11% mOS: 114 days	Terminated	[86]
Sabatolimab (MBG453)	Anti-TIM-3 monoclonal antibody	Azacitidine or decitabine	NCT03066648	Newly diagnosed HR and very HR-MDS *N* = 53	Phase Ib	ORR: 56.9% CR: 19.6% 12-m PFS: 51.9% mDoR: 16.1 m	Ongoing	[97]
		Placebo or HMA	NCT03946670	Patients with MDS, intermediate, HR, very HR Not eligible to transplant or intensive chemotherapy	Phase II STIMULUS-MDS1	No results yet	Ongoing	[98]
			NCT04266301	Patients with MDS, intermediate, HR, very HR or CMML-2 Not eligible to transplant or intensive chemotherapy	Phase III STIMULUS-MDS2	No results yet	Ongoing	[98]
		Azacitidine and venetoclax	NCT04812548	HR or very HR-MDS Not eligible for intensive chemotherapy or transplant	Phase II STIMULUS-MDS3	No results yet	Ongoing	[109]
CC-90002	Anti-CD47	monotherapy	NCT02641002	R/R AML (*N* = 24) and HR-MDS (*N* = 4)	Phase I CC-90002-AML-001	Patients with MDS, *N* = 4: SD in 2 patients 82% of the patients TD	Terminated	[100]
Magrolimab	Anti-CD47	Placebo or azacitidine	NCT04313881	Previously untreated HR-MDS	Phase III ENHANCE	No results yet	Ongoing	[105]

HMA: hypomethylating agents; MDS: myelodysplastic syndrome; Ipi: ipilimumab; Nivo: Nivolumab; AZA: azacitidine; PFS: progression-free survival ORR: overall response rate; CR: complete response; mOS: median overall survival; HR: high risk; CMML: chronic myelomonocytic leukemia; mDoR: median duration of response; and AML: acute myeloid leukemia; comb: combination; and m: months

### Monoclonal antibodies in MDS

One of the areas of immunotherapy research is the development of monoclonal antibodies and CAR T cell therapy in myeloid malignancies. The latter is another focus of development and will not be discussed here. There are three main classes of antibodies being studied in myeloid malignancies: antibody–drug conjugates (ADCs), bispecific T cell engagers (BiTEs), and immune checkpoint inhibitors (ICIs). ADCs are monoclonal antibodies conjugated with cytotoxic agents delivered once it attaches to the leukemic cell ^225^Ac-lintuzumab (CD33 radioimmunoconjugate), Iomab-B (CD45 radioimmunoconjugate), IMGN779 (CD33 ADC)—gemtuzumab ozogamicin, the first antibody–drug conjugate approved for induction therapy of AML, and IMGN632 (CD123 ADC). None of these, however, are approved for MDS. BiTEs bring T cells into proximity to leukemic cells being studied in many clinical trial but not yet approved for AML or MDS including AMG 330 (bispecific CD3/CD33 antibody), flotetuzumab, XmAb14045 (bispecific CD3/CD123 antibodies), MCLA-117 (bispecific CD3/CLL1 antibody), and AMV564 (bispecific CD3/CD33 T cell engager). Furthermore, many pathways can be targeted with ICIs (Fig. 1). The most studied are programmed cell death protein 1 (PD-1) (e.g., pembrolizumab, nivolumab) and programmed death-ligand 1 (PD-L1) (e.g., durvalumab, avelumab) inhibitors which act by inhibiting the association of PD-L1 with its receptor PD-1. These, together with the CTLA4 blocking antibody, ipilimumab, were the most tested in AML and MDS [77]. Some of the new coinhibitory pathways, such as TIM-3, LAG-3, TIGIT, and the macrophage ICIs CD47, are being explored in clinical trials. They display unique functions, particularly at tissue sites regulating different immunity features.

PD-1 and CTLA4 signaling has been involved in MDS pathogenesis and resistance mechanisms to HMAs. Yang et al*.* demonstrated an increased PD-1, PD-L1, PD-L2, and CTLA4 expression in peripheral blood mononuclear cells (PBMCs) from MDS patients under HMA treatment with AZA or AZA plus vorinostat, suggesting that resistance to AZA could be mediated by increased expression of these inhibitory markers [78]. Based on these data, an ipilimumab monotherapy trial was designed for HR-MDS patients who had failed HMAs. Twenty-nine patients received monotherapy induction at two dose levels (DL1 3 and DL2 10 mg/kg) every 3 weeks, followed by maintenance treatment for responders. Initially, a lot of immune-related adverse events (IRAEs) were seen with DL2, but these were not observed in the dose-expansion phase with DL1 with no grade 2–4 IRAEs reported in 18 additional patients. Best responses included mCR in only one patient (3.4%). Five patients underwent allo-HCT without excessive toxicity. Median survival for the group was 9.8 months (294 days, 95% CI: 240–671). Seven patients had prolonged stable disease (PSD) for ≥ 46 weeks (24% of all patients and 29% of those who received DL1), including 3 patients with PSD of > 1 year [79]. As observed in solid tumors, patients who respond to ICIs tend to have a prolonged response likely caused by biologic modulation of the disease. Other explanations could be that responders might have a more indolent disease and larger trials should be done to evaluate these findings. Analysis of PBMC samples in this study shows that patients achieving PSD or mCR had significantly higher proportion of CD4^+^ and CD8^+^ T cells expressing ICOS (inducible T cell costimulator) (*p* = 0.05 and *p* = 0.01, respectively), and TCR [79] compared to healthy donors. However, this did not correlate with clinical outcomes [81].

Ipilimumab was used for relapsed hematologic malignancies after allo-HSCT in a phase I/Ib study showing feasibility and tolerability. A total of 28 patients (AML = 12, Hodgkin lymphoma = 7, non-Hodgkin lymphoma = 4, MDS = 2) from six sites received induction therapy with ipilimumab for a total of 4 doses, with additional doses in patients who had clinical benefit. IRAE was observed in 6 patients (21%). Four patients (14%) developed graft-*versus*-host disease (GVHD) precluding further administration of ipilimumab. One death was observed. No responses were observed in patients who received the lower dose. Among 22 patients who received the higher dose, 5 (23%) had CR, 2 (9%) had PR, and 6 (27%) had decreased tumor burden. CR occurred in 4 patients with extramedullary AML and 1 with MDS developing into AML. Four patients had durable response for more than 1 year [80]. This trial completed accrual in June 2021, and final results are awaited (NCT01822509).

Another study from the MDACC group using nivolumab or ipilimumab with or without AZA in the frontline setting or after HMA failure showed more activity with ipilimumab. Frontline patients (*n* = 41) were treated with AZA in combination with nivolumab or ipilimumab. Patients in HMA failure group (*n* = 35) received single agent nivolumab or ipilimumab. The median follow-up was 20.1 months. Ipilimumab had more activity in the HMA failure setting with an ORR of 30%, far more significant than in the previous study (approximately 3%) but with similar median OS of 8.5 months compared to 9.8 months in the previous one [82]. These findings suggest that some patients with a particular BM environment could benefit from ICIs, and studies should focus on strategies to identify these patients and include them in ICIs trials.

One reason for ICIs failure is the increased number of myeloid-derived suppressor cells (MDSCs) in the BM of patients with MDS, which increases the immunosuppressive environment as the MDS progresses to AML. Preclinical data showed that the addition of entinostat (a histone deacetylase inhibitor) to PD-1 blockade significantly improves survival in knockout murine myeloid leukemia model, suggesting synergistic clinical activity in this setting [82]. In fact, resistance to PD-1 and PDL-1 seems to be mediated by MDSCs and targeting these cells with entinostat might restore ICIs activity with PD-1 blockade. Based on this data, a phase Ib trial was designed using the anti-PD-1 agent, pembrolizumab, in combination with entinostat after HMA failure (NCT02936752). Results of this ongoing multicenter trial in the USA are awaited.

Most of the ICIs studies have been single-center and small sample-sized studies. The FUSION-AML-001 trial was the first randomized trial assessing the use of ICIs in 84 previously untreated HR-MDS. Patients were randomized to standard HMA treatment (AZA) with or without the anti-PD-L1 mAb, durvalumab [83]. Although the CR rate in the durvalumab arm was numerically higher (36% *vs.* 19%), no statistically significant difference in ORR between treatment arms was observed (Arm A: 61.9% *vs.* Arm B: 47.6%, *p* = 0.184). Median OS and PFS were similar in both arms, 11.6 vs. 16.7 months and 8.7 vs. 8.6 months, respectively. These data may be indicative of that durvalumab might not be active in MDS but cannot be generalizable to all ICIs. Extensive correlative immune testing shows that AZA increased PD-L1 expression on the BM immune cells but not on the tumor blasts. Hence, PD-L1 expression on tumor blasts might not be an essential mechanism for resistance to HMA in this setting [83]. Yet, this theory warrants further study.

### Lenalidomide in patients with R/R HR-MDS or AML with trilineage dysplasia

Lenalidomide is an immunomodulatory drug studied in MDS aiming to manipulate the immune system. It also interferes with tumor microenvironment interactions through anti-angiogenesis activity, anti-inflammatory property by downregulating tumor necrosis factor α, downregulation of adhesion molecules, anti-proliferative, and anti-osteoclastogenic activity [84]. Lenalidomide is approved in MDS at a low dose of 5–10 mg in patients with del(5q) [85]. A study used high-dose lenalidomide (15 mg and 50 mg) in 27 patients with R/R HR-MDS (*n* = 16) and AML with trilineage dysplasia (*N* = 11). The 15 mg dose (first 9 patients) was well tolerated but with a 0% objective response. However, 50 mg (subsequent 18 patients) was hard to tolerate due to grade 3/4 non-hematologic toxicity (neutropenic infections), and only 39% received ≥ 2 cycles. The ORR was 11%. Thirty- and 60-day mortality rates were 15% and 30%, respectively, and only 5 patients survived ≥ 1 year. Median follow-up was 67 days. Median OS for the entire cohort was 114 days only (range, 15–841). The study was stopped and concluded that lenalidomide 50 mg appeared to be poorly tolerated and minimally active, and its use for R/R myeloid malignancies should be restricted to clinical trials [86]. Other trials also failed to show benefit of adding LEN to AZA in high-risk MDS, notably a phase II trial by the Nordic group [87].

### Sabatolimab (MBG453) in combination with HMAs in patients with HR-MDS

Sabatolimab (MBG453) is novel ICIs first-in-class investigational immuno-myeloid therapy that binds to TIM-3 [88, 89] (T cell immunoglobulin and mucin domain 3), a novel target expressed on macrophages, monocytes, NK cells, dendritic cells, and T cells. It is involved in regulating innate and adaptive immune responses [90, 91] and seems to be expressed on leukemic stem cells (LSCs) and blasts but not on hematopoietic stem cells (HSCs) [92, 93] making it a promising target in MDS/AML [88, 89, 91, 93–96]*.* In addition, it may inhibit TIM-3/galectin-9-driven LSC self-renewal via blockade of TIM-3 on LSCs [88, 89, 91–96].

Early data have suggested that sabatolimab has synergistic activity in combination with HMA. Hence, a dose-escalation phase Ib study was conducted, and results were presented at the 2021 ASH meeting [97]. The study comprised 48 patients with newly diagnosed AML, 53 with HR- and very HR-MDS and showed clinical activity of sabatolimab combined with an HMA (AZA or decitabine) with a high ORR of 56.9% in newly diagnosed HR and very HR-MDS patients. The mCR was 23.5%, and the CR rate was 19.6%, slightly higher than monotherapy (approximately 10–15%). The median DoR was 16.1 months and longer (21.5 months) in patients who achieved CR. The 12-month PFS was 51.9%, and 24% of these patients were able to proceed with allo-HSCT [97]. These results supported TIM-3 as a potential therapeutic target and provided a basis for the further development of sabatolimab + HMA in HR-MDS and AML. This resulted in a broad clinical trials program (STIMULUS) committed to evaluating sabatolimab in patients with myeloid malignancies [98]. One of these trials is the STIMULUS-MDS1 phase II trial (NCT03946670) of sabatolimab or placebo + HMA for very high-, high-, or intermediate (and ≥ 5% BM blasts at baseline)-risk MDS, which has completed recruitment , and results are awaited. The second study is STIMULUS-MSD2, a phase III trial (NCT04266301) of sabatolimab or placebo + AZA for very high- or intermediate-risk MDS or CMML-2, also fully enrolled 530 patients, and results are pending. The third trial, STIMULUS-MDS3, is a single-arm phase II trial (NCT04812548) with sabatolimab in combination with VEN and AZA [98].

### Anti-CD47 antibody in Subjects with relapsed/refractory HR-MDS

CD47 is a dominant macrophage checkpoint with a “don’t eat me” signal, overexpressed in myeloid malignancies which leads to tumor evasion of phagocytosis by macrophages. Blockade of CD47 leads to engulfment of leukemic cells and therapeutic elimination [99]. Clinical studies have been underway with CD47 targeting agents in AML and MDS as monotherapy and in combination. One of these trials is CC-90002 dose-escalation study which included patients with R/R AML (*n* = 24) and HR-MDS (*n* = 4) and used anti-CD47 as monotherapy. Unfortunately, responses were not observed, mainly due to anti-drug antibody development in most patients, and the study was discontinued. The monotherapeutic design may have contributed to this negative result [100]. Hence, CD47 blockade was tested in combination with other antineoplastic agents, namely AZA and ruxolitinib. Preclinical studies showed that combination of anti-CD47 drugs with AZA significantly enhances phagocytosis leading to the elimination of AML cells by human macrophages in vitro and improves clearance of AML in vivo. The combination prolonged survival compared to single agent [101]. In fact, treatment with AZA has shown to upregulated (four–sixfold) CD47 expression in MDS/MPN cell line models. This led to cancer cell survival and resistance to treatment [102].

This combination tested with another similar ICIs is the anti-CD47 mAb magrolimab that has led to significant reductions in blasts both in AML and MDS when combined with AZA. Magrolimab plus AZA combination showed promising results in AML with an ORR of 65% and 44% CR rate with similar responses in the subgroup of patients with TP53 mutation (ORR 71% and CR rate 48%) [103]. In fact, *TP53* mutations have been shown to be associated with significant immune dysregulation and an immunosuppressive environment. This is coupled with an increased expression of inhibitory immune molecules such as PDL-1, partly explaining why patients with *TP53* mutations are more susceptible to ICIs. Notably, PD-L1 expression is significantly increased in *TP53*-mutated AML/MDS, which is associated with MYC upregulation and marked downregulation of its negative regulator miR-34a (a p53 transcription target) compared to the *TP53* wild-type disease [104]. The phase III ENHANCE trial is currently assessing the use of magrolimab in previously untreated HR-MDS in combination with AZA. The results will add to our understanding of how this agent fits into treatment regimens [105].

## Conclusion

The landscape of the management of patients with HR-MDS has changed with the introduction of HMAs. They showed to improve hematopoiesis and quality of life and, in the case of AZA, prolonged survival as demonstrated in a large randomized trial. However, multiple real-life and registry analyses have demonstrated minimal improvement in survival at the population level after the approval of HMAs. Furthermore, the 24-month median survival observed with AZA in the landmark AZA-001 trial has not been replicated in population-based studies or other clinical trials using AZA monotherapy arms. In conclusion, several active agents are being tested in clinical trials (some of the more exciting ones being sabatolimab, VEN, IDH inhibitors, and magrolimab). Many others are also being tested. In parallel with research into novel agents, we must always consider and encourage frontline clinical trial participation and discuss this with every newly diagnosed patient with HR-MDS rather than defaulting to the routine use of HMAs. We must face the challenges with wider-scale enrollment in frontline HR-MDS clinical trials and suggest solutions to accelerate this process with the goal of achieving a real and substantial change in the natural history of this aggressive malignancy [12, 13, 106]. Also, the endpoints of clinical trials should focus on duration of response and ultimately survival rather than overall and complete response rates. Finally, the determination of patients’ risk is critical in selecting the optimal therapeutic approach in the era of precision medicine. The recent development of an MDS risk stratification model that incorporates molecular abnormalities into its risk strata, the molecular international prognostic scoring system, will undoubtedly help shape the disease, hence improving treatment choice and inclusion of patients in clinical trials [107].

## Data Availability

Not applicable.
